# Transcriptional Profiling of Ileocecal Valve of Holstein Dairy Cows Infected with *Mycobacterium avium* subsp. *Paratuberculosis*

**DOI:** 10.1371/journal.pone.0153932

**Published:** 2016-04-19

**Authors:** Randy J. Hempel, John P. Bannantine, Judith R. Stabel

**Affiliations:** USDA-Agricultural Research Service (ARS), National Animal Disease Center, Ames, Iowa, United States of America; Indian Institute of Technology Delhi, INDIA

## Abstract

Johne’s disease is a chronic infection of the small intestine caused by *Mycobacterium avium* subspecies *paratuberculosis* (MAP), an intracellular bacterium. The events of pathogen survival within the host cell(s), chronic inflammation and the progression from asymptomatic subclinical stage to an advanced clinical stage of infection, are poorly understood. This study examines gene expression in the ileocecal valve (ICV) of Holstein dairy cows at different stages of MAP infection. The ICV is known to be a primary site of MAP colonization and provides an ideal location to identify genes that are relevant to the progression of this disease. RNA was prepared from ICV tissues and RNA-Seq was used to compare gene transcription between clinical, subclinical, and uninfected control animals. Interpretation of the gene expression data was performed using pathway analysis and gene ontology categories containing multiple differentially expressed genes. Results demonstrated that many of the pathways that had strong differential gene expression between uninfected control and clinical cows were related to the immune system, such as the T- and B-cell receptor signaling, apoptosis, NOD-like receptor signaling, and leukocyte transendothelial migration pathways. In contrast, the comparison of gene transcription between control and subclinical cows identified pathways that were primarily involved in metabolism. The results from the comparison between clinical and subclinical animals indicate recruitment of neutrophils, up regulation of lysosomal peptidases, increase in immune cell transendothelial migration, and modifications of the extracelluar matrix. This study provides important insight into how cattle respond to a natural MAP infection at the gene transcription level within a key target tissue for infection.

## Introduction

Johne’s disease (paratuberculosis), a chronic granulomatous enteritis of ruminant animals, is caused by *Mycobacterium avium* subsp. *paratuberculosis* (MAP). Johne’s disease continues to have major economic and animal welfare consequences, and remains a serious problem both nationally and internationally [1, 2]. Transmission of MAP primarily occurs by fecal-oral transmission or by consumption of milk from infected dams in young calves. After infection, the pathogen is taken up by either M-cells in Peyer’s patches in the distal ileum or through epithelial cells in the ileum and ileocecal valve [3–5]. After crossing the intestinal epithelium, MAP is phagocytosed by antigen presenting cells where it establishes a chronic infection in susceptible animals.

One of the major contributing factors to the prevalence of Johne’s disease throughout the world is due in part to the difficulty of identifying infection before the bacterium is shed and transmitted to herd mates. Cattle may remain asymptomatic for extended periods of time, approximately 2–5 years, while shedding low amounts of MAP in their feces. During the subclinical stage, animals remain very difficult to diagnose and the disease may progress further to a more advanced clinical stage, when the outward signs of disease appear. The terminal, clinical stage is often characterized by high shedding of MAP in the feces, chronic diarrhea, rapid weight loss, decreased milk production, diffuse edema, and infertility [6]. At this stage of disease, the producer typically culls the animals due to losses in productivity.

The primary defense of the host to prevent MAP infection is the innate immune system, including physical barriers such as the thick mucous layer lining the intestine, epithelial cell tight junctions, as well as antimicrobial peptides. Macrophages and dendritic cells are key immune cells involved in removing pathogenic bacteria from the host early in infection. However, MAP has developed several mechanisms that allow it to survive and replicate within macrophages, primarily by inhibiting the maturation of the phagosome [7]. MAP has been found to further inhibit macrophage function via secretion of a lipid phosphatase that prevents the acquisition of lysosomal components to the phagosome [8]. These mechanisms provide support for the idea that MAP is able to manipulate the macrophage and establish a suitable environment for MAP to survive and replicate.

Understanding the adaptive immune response to MAP infection is critical for controlling the spread of this disease since cross-talk between macrophages and T cells is key to cellular activation and maintenance of an active immune response to rid the host of the pathogen. Several *in vitro* and *in vivo* analyses of the immune response to MAP have given insight into the interaction between the host and pathogen [9, 10]. During the early stages of infection, a strong T_H_1 response is observed, with IFN-**γ** being the primary effector cytokine. However, later in the infection there is a transition from T_H_1 to T_H_2-mediated responses, with a subsequent rise in MAP-specific antibodies. It is believed that a T_H_2 response is less effective at controlling MAP infection, which allows the infection to progress into the more severe clinical stage of disease.

The aim of this study is to analyze the transcriptomes of cattle at different stages of Johne’s disease to identify the host response during progression of the disease. Differentially expressed (DE) genes were identified by comparing the transcription level of genes from the ileocecal valves of uninfected controls, subclinical, and clinical Holstein dairy cows. These genes were further analyzed using the DAVID Functional Annotation tool to identify gene ontology groups and cellular pathways underlying natural MAP infection. We discovered that the cow ramps up metabolism early in infection and switches to immune defenses as the infection is more established.

## Materials and Methods

### Animals

Naturally infected and uninfected control Holstein dairy cows were purchased from dairies in Minnesota and Iowa. Producer names cannot be disclosed due to confidentiality agreements. Cows were then housed at the National Animal Disease Center (NADC, Ames, IA) for varying periods of time. Cows were monitored on a semi-annual basis by obtaining blood and fecal samples to assess disease status while they were maintained on-site. Uninfected control animals were housed separately from MAP infected animals at all times. Animals were classified as uninfected control (n = 5), subclinical, (n = 5) or clinical (n = 5) based upon measurable MAP fecal shedding and serologic assessment of status by whole blood IFN-**γ** (Bovigam, Thermo Fisher, Waltham, MA) and Herdchek ELISA (IDEXX Laboratories, Westbrook, ME). In addition, status of cows was also characterized by quantitative culture of MAP and histopathologic scoring of acid fast stain and lesion severity in the ICV, as well as other sections of the small intestine obtained at the time of euthanasia. All control animals were negative for MAP in the feces and tissues by culture and acid fast staining. Subclinical animals were classified by shedding ≤ 10 CFU/gram of feces or tissues, whereas, clinical animals were classified by having > 30 CFU/gram of feces or tissues at time of euthanasia. Lesion scores were obtained for all sections of small intestine (ileum, jejunum, ileocecal valve, and ileocecal lymph node) and were ranked on a scale of 0 (no acid fast bacteria) to 5 (acid fast bacteria positive and severe granulomatous lesions consistent with MAP infection). Criterion for the stratification of cows used in this study is shown in Table 1. The USDA-ARS-National Animal Disease Center Institutional Animal Care and Use Committee approved all procedures performed on the animals used in this study, and animal comfort was monitored carefully by caretaker and veterinarian staff.

**Table 1:** Animal infection status classifications.

Infection Status	Cow ID	MAP CFU/ gram of tissue	MAP CFU/ gram of feces	Lesion Scores
Uninfected Control	0017	0	0	0
	0018	0	0	0
	0441	0	0	0
	0490	0	0	0
	8102	0	0	0
Subclinical	0030	0	0	0
	0072	0	0	0
	0787	7.50E+02	0	0
	1061	4.25E+02	0	0
	1145	0	0	0
Clinical	0010	TNTC	TNTC	4
	0035	TNTC	TNTC	3
	0041	TNTC	TNTC	3
	0789	TNTC	TNTC	4
	5545	TNTC	TNTC	3

TNTC = Too numerous to count.

### Total RNA extraction

RNA was extracted from ileocecal valve tissues from Holstein dairy cows. Tissues were rinsed in PBS, snap frozen in liquid nitrogen and stored at –80°C until time of processing. Tissues were from uninfected controls, asymptomatic cows with subclinical disease, and symptomatic cows with clinical disease. Approximately 200 mg of frozen tissue was removed using a sterile scalpel and placed in 2.0 mls of RNAlater-ICE (Life Technologies) and stored at -80°C according to manufacturer’s protocol. Prior to RNA isolation, tissues in RNAlater-ICE were moved to -20°C for approximately 24 hours. Tissues were removed from -20°C and placed on ice. Approximately 50 mg of tissue was removed from RNAlater-ICE and placed in gentleMACS M tubes (Miltenyi Biotec, Bergisch Gladbach, Germany) containing ice cold RLT Plus buffer (Qiagen, Hilden, Germany) with β-mercaptoethanol (diluted 1:100). Tissues were homogenized using the gentleMACS Octo Dissociator (Miltenyi Biotec, Bergisch Gladbach, Germany), followed by centrifugation at 2000 x g for 5 minutes to remove tissue debris. The supernatant was carefully removed and used for RNA extraction using the RNeasy Mini Plus Kit (Qiagen). An on-column DNase digest, using RNase-free DNase I (Qiagen), was used to remove any residual DNA. The absence of DNA was confirmed by PCR.

### RNA-Seq Mapping and Gene Expression Analyses

Approximately 4 μg of total RNA was used for library creation. To insure RNA integrity, each sample was assessed using the Agilent RNA 6000 Nano kit with the Agilent 2100 Bioanalyzer (Agilent Technologies, Santa Clara, CA). Only RNA samples with an RNA integrity number (RIN) greater than 8 (an indication of high quality RNA with little degradation) were used for RNA-seq. The RNA-seq library was prepared using the mRNA Seq Single Read Library preparation kit per manufacturer’s instructions (Illumina Inc., San Diego, CA). cDNA libraries were sequenced using Illumina sequencing technology (Illumina HiSeq 2500) in five lanes of the sequencing flow cell. Libraries were prepared and sequenced at the Iowa State University DNA Facility (Ames, IA). Sequence data were submitted to the National Center for Biotechnology Information Sequence Read Archive under Accession No. SRA279163.

Sequences were analyzed for quality using FastQC [version 0.10.1] (http://www.bioinformatics.babraham.ac.uk/projects/fastqc/) revealing some sequences with low quality scores at the 3’ end. Adaptors and low quality regions were trimmed using prinseq-lite [11]. Trimmed reads were aligned to the *Bos taurus* genome (Ensembl UMD3.1 version 70) with TopHat splice junction mapper [12] [version 2.0.11] which aligns reads using the Bowtie method [13] [version 2.2.2]. SAMtools [version 0.1.19] [14] was used to sort the accepted alignments BAM file prior to using the HTSeq package [version 0.6.1p1] [15] to obtain raw counts per transcript. All uniquely mapped reads were counted for each bovine Ensembl gene and transcript. Picard Tools was used to analyze the location of sequence reads with respect to known genomic regions (http://broadinstitute.github.io/picard). Differential expression analysis was performed on the raw counts in the R statistical programming environment [16] using the EdgeR [version 3.8.6] Bioconductor package [17, 18]. DAVID Function Annotation tool was used to identify pathways with multiple DE genes (http://david.abcc.ncifcrf.gov) [19, 20].

## Results

### Summary of RNA-seq data

**Fig 1:**
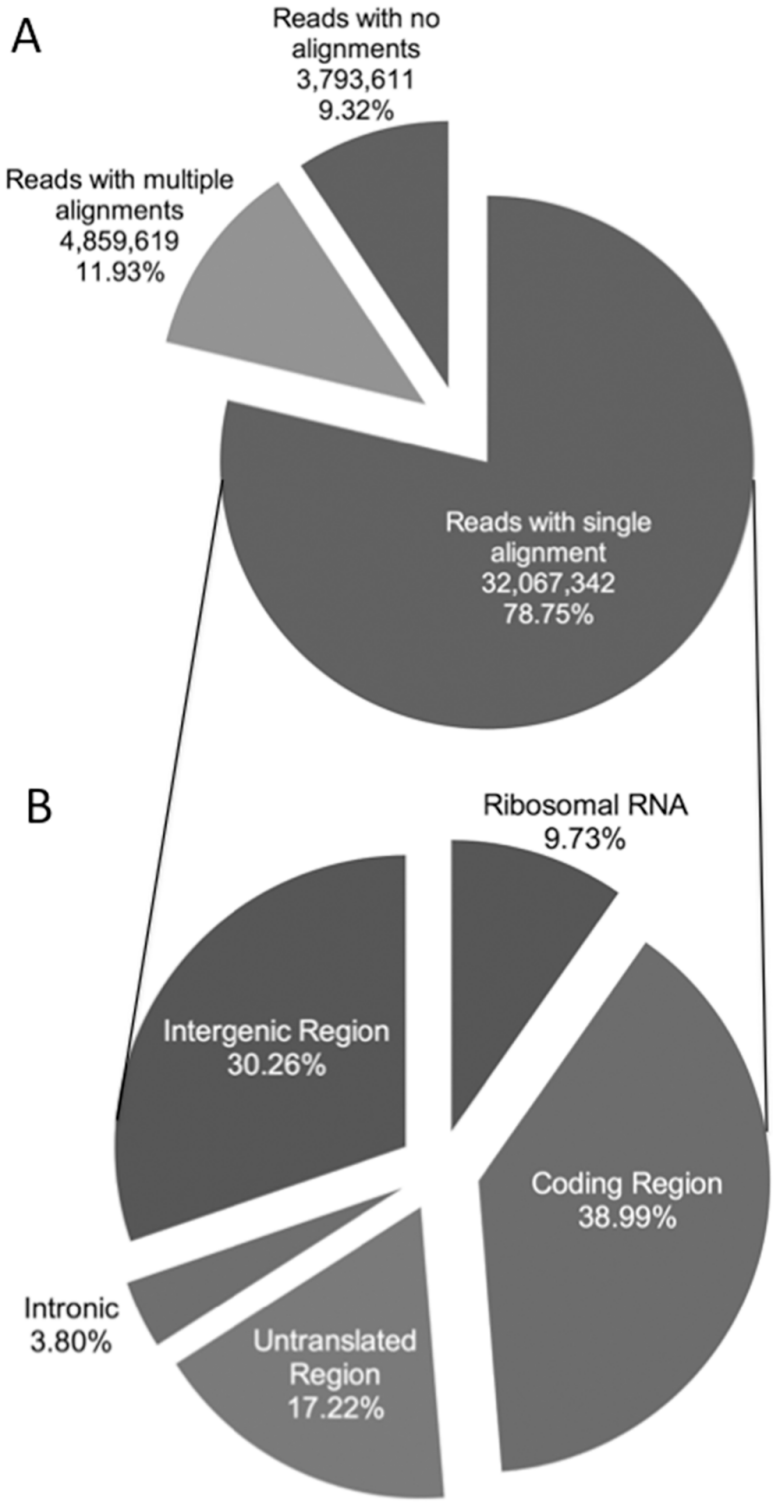
Description of reads mapping to single and multiple locations in the *B*. *taurus* genome. A) Pie chart showing the mean number and percentage of reads that aligned to the reference genome using TopHat and Bowtie. B) Pie chart showing the mean percentage of uniquely mapped reads assigned to coding regions (i.e. known exons), untranslated regions (i.e. 3’ or 5’ untranslated regions), intronic regions (i.e. known introns), intergenic regions (i.e. areas where no known annotated genes are present), and ribosomal RNA based on alignment data from HTSeq and Piccard Tools.

An RNA-seq library was prepared for each animal within the three health stages, uninfected control, subclinical and clinical cows, resulting in 5 RNA-seq libraries for each group. All the libraries were sequenced within 5 lanes of one Illumina flow cell, generating an average of 122.2 million reads per lane. The average number of reads per sample was 40.7 million (Fig 1A). Alignment of the RNA-seq reads to the *B*. *taurus* genome (Ensembl UMD 3.1) yielded a mean of 36.9 million reads per sequenced library. An average of 32.1 million reads aligned to unique locations. Approximately 4.39 million reads aligned to multiple locations within the genome and were excluded from the gene expression analysis. A more detailed analysis of the reads mapping to unique locations revealed that 39% aligned to coding regions while 17.2% aligned to untranslated regions (UTR) of mRNAs (Fig 1B). Ribosomal genes accounted for 9.73% of reads and 30.3% of aligned reads mapped to intergenic regions.

**Fig 2:**
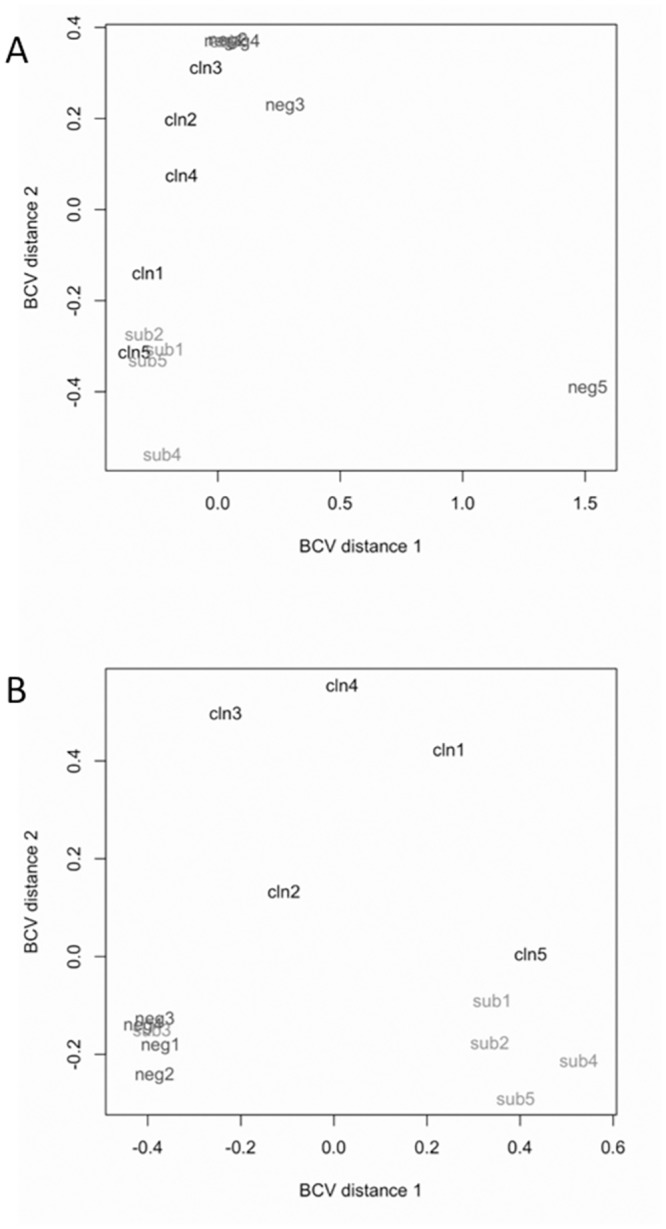
Multidimensional scaling plots of samples from uninfected control, subclinical, and clinical animals based on RNA-seq data. Distance 1 and distance 2 separate all samples based on the expression values of all 12,133 genes that passed filtering criteria prior to differential gene expression analysis. A) Comparison of all 15 samples. B) Comparison of 14 samples after the removal of uninfected control animal 5 (Cow #8102).

HT-Seq, a program designed to process high-throughput sequencing data, was used to quantify the reads that aligned to the bovine Ensembl gene IDs. The genes were further filtered to remove genes that didn’t have at least 1 read per million mapped in seven or more animals, which yielded 12,132 genes used in downstream analyses. Prior to differential gene expression analysis using EdgeR, the normalized count data were used to generate a multidimensional scaling (MDS) plot to observe sample clustering and to identify outliers (Fig 2). One cow in the uninfected control group (#8102) did not cluster closely with any group and was removed in downstream analyses (Fig 2A). Outliers were expected since these cows were not age matched and were acquired from farms outside of our facility, where the cows were naturally infected with MAP. However, despite all the animals coming from different outside sources at different stages of disease, the samples differentiated according to their disease status (Fig 2B).

### Differential Gene Expression Analysis

**Fig 3:**
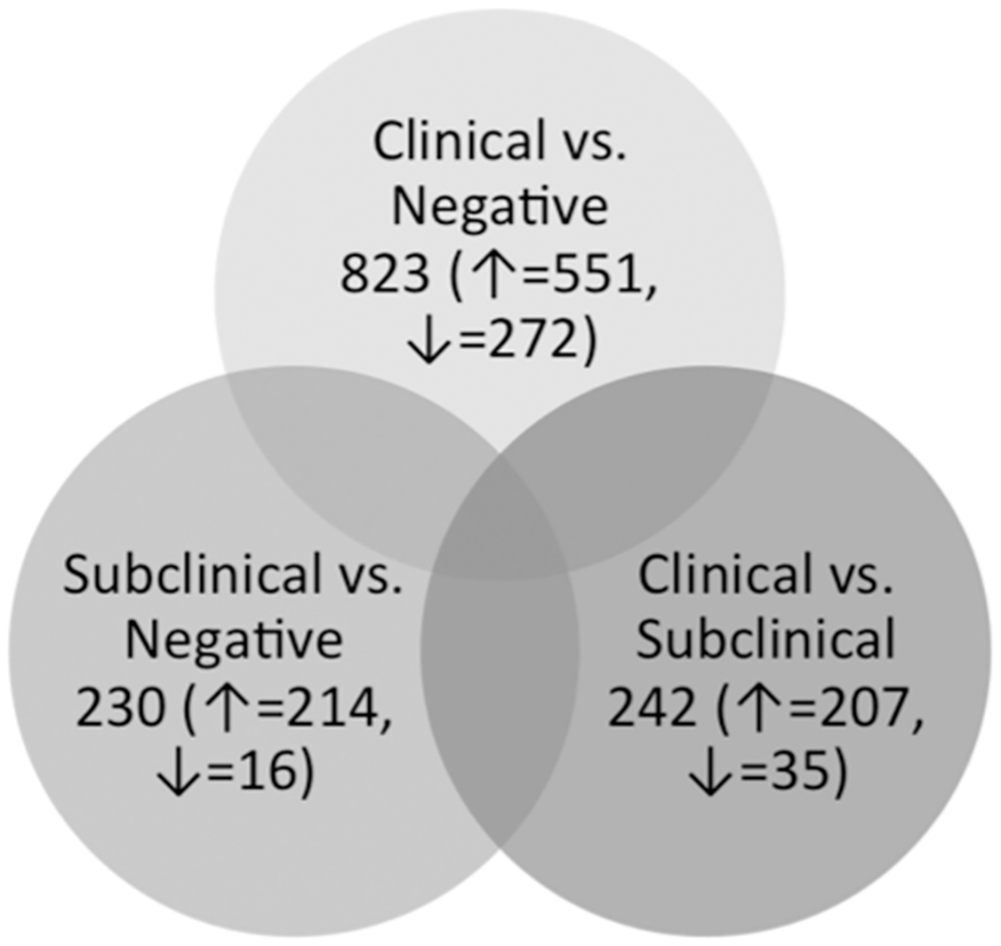
Venn diagram showing the comparison of differentially expressed genes between all group comparisons. Venn diagram showing the number of DE genes in each group comparison as well as the numbers of genes that are up- (↑) or down-regulated (↓) in each dataset.

A systematic comparison was performed between each of the groups of animals based on their disease status. Statistical analysis of all expressed genes resulted in 823, 242, or 230 DE genes (FDR threshold of ≤ 0.05) in the group comparisons between uninfected control and clinical cows, subclinical and clinical cows, and uninfected control and subclinical cows, respectively (Fig 3). These comparisons also resulted in many DE genes that are common between two different groups, as well as a total of eight genes that are common amongst all groups. All DE genes were further subdivided into groups depending upon the direction of their regulation (up- or down-regulated). It is important to note that in each of the group comparisons, there were markedly more up-regulated genes compared to down-regulated genes, suggesting that MAP infection results in increased transcriptional activity rather than inhibition of transcriptional activity.

### Gene Expression Comparison of Clinical vs. Uninfected Control Animals

The most DE genes were identified in the clinical versus uninfected control animal tissues. This makes biological sense because these conditions represent the opposite ends of the disease spectrum with subclinical as the intermediary stage. A total of 823 DE genes were identified in this comparison (FDR ≤ 0.05), of which 551 were up-regulated and 272 genes were down-regulated in the clinical animals compared to the uninfected controls.

The top up-regulated genes based on the fold change of expression consisted of the fatty acid binding protein 6 (*FABP6*), solute carrier family 10 member 2 (sodium/bile acid cotransporter) (*SLC10A2*), matrix metallopeptidase 13 (*MMP13*), apolipoprotein B (*apoB*), and the immunoglobulin superfamily member 23 (*IGSF23*). Genes that were down-regulated between clinical and uninfected control cows were granulysin-like (*GNLY*), Fc receptor-like A (*FCRLA*), uncharacterized protein containing membrane-spanning 4-domains, subfamily A; member 1 (*MS4A1*), an uncharacterized protein (ENSBTAG00000013039), and CD79b molecule (Table 2).

**Table 2:** Gene regulation of top regulated genes between clinical and uninfected control animals.

Gene ID	Description	Log_2_ Fold Change	P-value	FDR
ENSBTAG00000010632	fatty acid binding protein 6, ileal (FABP6)	7.33	2.10E-05	1.56E-03
ENSBTAG00000005925	solute carrier family 10 (sodium/bile acid cotransporter), member 2 (SLC10A2)	6.25	9.69E-06	8.97E-04
ENSBTAG00000015059	matrix metallopeptidase 13 (collagenase 3) (MMP13)	5.70	2.53E-10	1.48E-07
ENSBTAG00000008505	apolipoprotein B	5.63	7.91E-08	2.04E-05
ENSBTAG00000048075	immunoglobulin superfamily, member 23	5.60	3.25E-07	6.55E-05
ENSBTAG00000044204	CD79b molecule, immunoglobulin-associated beta	-2.97	9.65E-05	4.39E-03
ENSBTAG00000013039	Uncharacterized protein	-3.21	4.16E-04	1.18E-02
ENSBTAG00000004580	membrane-spanning 4-domains, subfamily A, member 1 (MS4A1)	-3.31	8.19E-05	4.01E-03
ENSBTAG00000034159	Fc receptor-like A	-3.76	2.52E-05	1.80E-03
ENSBTAG00000031828	granulysin-like (LOC616323) (GNLY)	-4.01	3.34E-04	1.00E-02

FDR = False Discovery Rate

**Fig 4:**
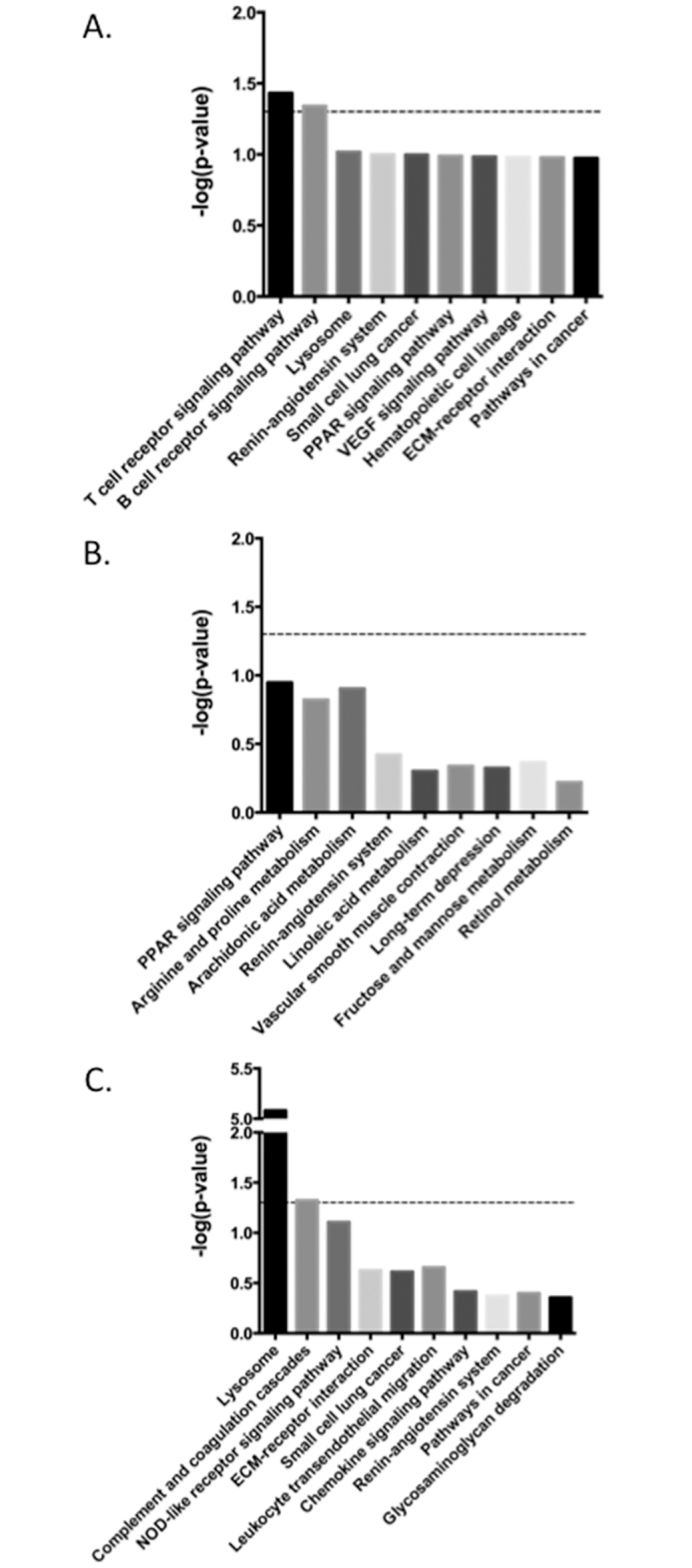
Top 10 functional networks that are modulated in group comparisons of differentially expressed genes. Functional networks that are modulated in the comparison between A) clinical and uninfected control animals, B) subclinical and uninfected control animals, C) clinical and subclinical animals.

**Fig 5:**
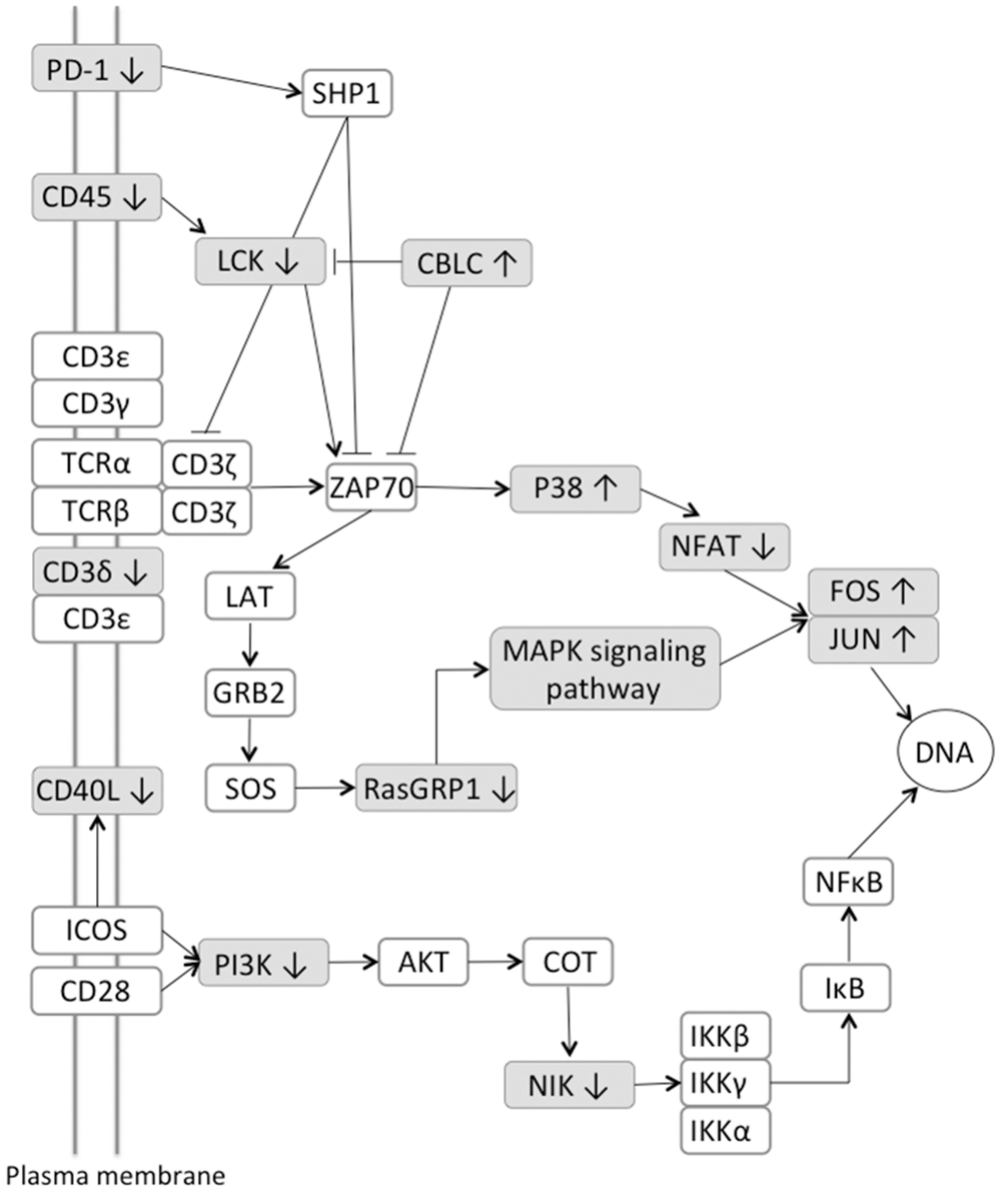
Pathway for T-cell receptor signaling for gene regulation comparison between clinical and uninfected control animals. Genes associated with TCR signaling that are differentially expressed are shaded in grey; genes with a white background represent genes that were not significantly differentially expressed.

Pathway analysis was performed on all 823 DE genes using the DAVID Functional Annotation tool. This identified an enrichment of genes involved in T-cell and B-cell receptor signaling and the lysosomal pathways (Fig 4A, Table 3). Within the T-cell receptor pathway genes that were differentially expressed included *MAPK13/p38*, *CD3δ*, *CD40LG*, *CD45*, *CBLC*, *LCK*, *MAP3K14/NIK*, and *PD–1*. There are a total of 16 DE genes in the T-cell receptor signaling pathway, 11 of which were down-regulated and 5 that were up-regulated in the clinical cows compared to the uninfected control group (Fig 5). There were several DE genes that are common between the T-cell and B-cell receptor signaling pathways. Of the 11 DE genes observed within the B-cell receptor pathway, 3 DE genes were unique: *SHIP/INPP5D*, *Igα/CD79A*, and *Igβ/CD79B*. It is of special note that the *Igα* and *Igβ* genes were highly down-regulated in the clinical cows compared to the uninfected controls, as both the T-cell receptor and B-cell receptor signaling pathways are critical for modulating host immune function.

**Table 3:** Regulation of DE genes involved in relevant pathways between clinical and uninfected control animals.

Gene ID	Description	Log_2_ Fold Change	P-value	FDR
**T-Cell Receptor Pathway**				
ENSBTAG00000004322	FBJ murine osteosarcoma viral oncogene homolog (FOS)	2.42	1.78E-11	1.55E-08
ENSBTAG00000010007	mitogen-activated protein kinase 13 (MAPK13/p38δ)	0.99	3.73E-04	1.08E-02
ENSBTAG00000004037	jun proto-oncogene (JUN)	0.85	1.52E-03	2.90E-02
ENSBTAG00000009494	Cas-Br-M (murine) ecotropic retroviral transforming sequence c (CBLC)	0.73	1.41E-03	2.77E-02
ENSBTAG00000013851	B-cell CLL/lymphoma 10 (BCL10)	0.69	1.76E-03	3.18E-02
ENSBTAG00000007084	mitogen-activated protein kinase kinase kinase 14 (MAP3K14) (NIK)	-0.69	5.15E-04	1.38E-02
ENSBTAG00000006909	phosphatidylinositol-4,5-bisphosphate 3-kinase, catalytic subunit beta	-0.79	2.29E-03	3.86E-02
ENSBTAG00000018984	phosphatidylinositol-4,5-bisphosphate 3-kinase, catalytic subunit delta	-0.97	1.00E-03	2.18E-02
ENSBTAG00000023144	protein tyrosine phosphatase, receptor type, C	-0.97	3.08E-04	9.56E-03
ENSBTAG00000006452	CD3d molecule, delta (CD3-TCR complex) (CD3D)	-1.00	3.07E-03	4.72E-02
ENSBTAG00000012695	lymphocyte-specific protein tyrosine kinase (LCK)	-1.05	1.64E-03	3.05E-02
ENSBTAG00000014698	caspase recruitment domain family, member 11 (CARD11)	-1.10	1.57E-04	6.13E-03
ENSBTAG00000021872	RAS guanyl releasing protein 1 (calcium and DAG-regulated) (RASGRP1)	-1.11	7.24E-04	1.74E-02
ENSBTAG00000018270	nuclear factor of activated T-cells, cytoplasmic, calcineurin-dependent 2 (NFAT)	-1.19	6.33E-04	1.57E-02
ENSBTAG00000017843	CD40 ligand (CD40LG)	-1.45	2.05E-03	3.57E-02
ENSBTAG00000011543	programmed cell death 1 (PDCD1)	-1.63	2.82E-03	4.45E-02
**B-Cell Receptor Pathway**				
ENSBTAG00000004322	FBJ murine osteosarcoma viral oncogene homolog (FOS)	2.42	1.78E-11	1.55E-08
ENSBTAG00000004037	jun proto-oncogene (JUN)	0.85	1.52E-03	2.90E-02
ENSBTAG00000013851	B-cell CLL/lymphoma 10 (BCL10)	0.69	1.76E-03	3.18E-02
ENSBTAG00000006909	phosphatidylinositol-4,5-bisphosphate 3-kinase, catalytic subunit beta	-0.79	2.29E-03	3.86E-02
ENSBTAG00000018984	phosphatidylinositol-4,5-bisphosphate 3-kinase, catalytic subunit delta	-0.97	1.00E-03	2.18E-02
ENSBTAG00000014698	caspase recruitment domain family, member 11 (CARD11)	-1.10	1.57E-04	6.13E-03
ENSBTAG00000020173	inositol polyphosphate-5-phosphatase, 145kDa (INPP5D)	-1.18	1.63E-04	6.30E-03
ENSBTAG00000018270	nuclear factor of activated T-cells, cytoplasmic, calcineurin-dependent 2	-1.19	6.33E-04	1.57E-02
ENSBTAG00000008006	RAS guanyl releasing protein 3 (calcium and DAG-regulated) (RASGRP3)	-1.56	9.89E-04	2.17E-02
ENSBTAG00000001882	CD79a molecule, immunoglobulin-associated alpha (CD79A)	-2.35	3.84E-05	2.36E-03
ENSBTAG00000044204	CD79b molecule, immunoglobulin-associated beta	-2.97	9.65E-05	4.39E-03
**Lysosomal Pathway**				
ENSBTAG00000021035	cathepsin K (CTSK)	4.01	4.62E-13	6.23E-10
ENSBTAG00000000133	CD68 molecule (CD68)	2.40	3.26E-07	6.55E-05
ENSBTAG00000010992	cathepsin H (CTSH)	1.63	3.81E-05	2.36E-03
ENSBTAG00000003636	lipase A, lysosomal acid, cholesterol esterase	1.54	9.10E-05	4.27E-03
ENSBTAG00000017465	glucosamine (N-acetyl)-6-sulfatase (GNS)	1.50	1.96E-06	2.67E-04
ENSBTAG00000012442	cathepsin B (CTSB)	1.49	8.69E-05	4.17E-03
ENSBTAG00000018784	cathepsin Z (CTSZ)	1.13	1.65E-04	6.33E-03
ENSBTAG00000011100	cathepsin C (CTSC)	1.04	1.33E-03	2.66E-02
ENSBTAG00000017077	cathepsin L2 (CTSL2)	0.94	2.48E-03	4.05E-02
ENSBTAG00000017135	cathepsin S (CTSS)	0.94	9.33E-04	2.09E-02
ENSBTAG00000015512	hexosaminidase B (beta polypeptide) (HEXB)	0.92	1.18E-04	5.02E-03
ENSBTAG00000008341	arylsulfatase B (ARSB)	-1.18	1.47E-04	5.87E-03
ENSBTAG00000006552	lysosomal-associated membrane protein 3 (LAMP3)	-1.22	9.80E-05	4.42E-03
ENSBTAG00000006780	napsin A aspartic peptidase	-2.10	2.32E-03	3.90E-02

There were also several DE genes that are part of the apoptotic pathway. Of the 82 genes that are annotated as having a role in apoptosis in the *B*. *taurus* genome, 9 genes were found to be differentially expressed. One of the up-regulated DE genes in this pathway included the death receptor tumor necrosis factor receptor superfamily, member 1A (*TNFRSF1A*), which was up-regulated in clinical animals. Another member of the apoptosis pathway that was up-regulated in clinical disease was IL-1β, along with several key proteases including caspases 3 and 7 (*CASP3/7*), and calpain 2 (*CAPN2*).

### Gene Expression Analysis in Subclinical vs. Uninfected Control Animals

Analysis of the RNA-seq reads in the subclinical animals and uninfected controls identified 230 DE genes. There was a large disparity between the up- and down-regulated genes; with 214 up-regulated and only 16 down-regulated genes in the comparison between these 2 treatment groups. The top 5 up-regulated genes based upon fold change between groups were: carbamoyl-phosphate synthase 1 (*CPS1*), carboxypeptidase O (*CPO*), tubulointerstitial nephritis antigen (*TINAG*), solute carrier family 7, member 9 (*SLC7A9*), and solute carrier family 6, member 19 (*SLC6A19*). The top 5 down-regulated genes were: WAP four-disulfide core domain protein 18 precursor (*WFDC18*), cadherin 16 (*CDH16*), annexin A8-like 1 (*ANXA8L1*), and two uncharacterized proteins (ENSBTAG00000047121 and ENSBTAG00000047740) (Table 4). There were large differences in the amount of regulation between the up- and down-regulated genes with the top 5 up-regulated and down-regulated genes averaging a 6.7 and -2.4 log-fold-change, respectively.

**Table 4:** Top regulated genes between subclinical and uninfected control animals.

Gene ID	Description	Log2 Fold Change	PValue	FDR
ENSBTAG00000016662	carbamoyl-phosphate synthase 1, mitochondrial (CPS1)	7.04	2.17E-06	5.37E-04
ENSBTAG00000019190	carboxypeptidase O (CPO)	6.99	6.42E-08	5.56E-05
ENSBTAG00000012652	tubulointerstitial nephritis antigen (TINAG)	6.54	1.82E-07	9.58E-05
ENSBTAG00000013338	solute carrier family 7 (glycoprotein-associated amino acid transporter light chain, bo,+ system), member 9 (SLC7A9)	6.42	7.11E-07	2.54E-04
ENSBTAG00000045695	solute carrier family 6 (neutral amino acid transporter), member 19	6.34	6.61E-07	2.51E-04
ENSBTAG00000018499	annexin A8-like 1 (ANXA8L1)	-1.80	2.10E-03	8.69E-02
ENSBTAG00000047121	Uncharacterized protein	-1.95	1.01E-03	5.22E-02
ENSBTAG00000009585	cadherin 16, KSP-cadherin (CDH16)	-2.38	2.82E-04	2.08E-02
ENSBTAG00000047740	Uncharacterized protein	-2.86	4.19E-03	1.32E-01
ENSBTAG00000025260	WAP four-disulfide core domain protein 18 precursor	-3.22	5.38E-04	3.42E-02

Contrary to the pathway analysis performed on the uninfected control and the clinical animals, where several pathways were related to the immune system, the analysis performed on the uninfected control and subclinical animal data revealed that metabolic pathways were the most affected. Out of the top 10 pathways identified by the DAVID Functional Annotation tool, 7 of them were metabolic pathways (Fig 4B). No pathways related to the immune system were identified in this comparison, indicating the animal may already be able to control an infection during the subclinical stage. This is contrary to the comparisons with the clinical animals where the majority of pathways with DE genes are related to immunological responses.

### Gene Expression Analysis in Clinical vs. Subclinical Animals

There are a total of 242 DE genes that were identified by edgeR between the subclinical and clinical animals. There is a large difference between the number of up- and down-regulated genes in this group comparison, 207 and 35, respectively. However, the level of gene expression changes was similar between up- and down-regulated genes as demonstrated by the log_2_ fold-changes. The top 5 up-regulated genes on average demonstrated a 4.0-fold increase between clinical and subclinical cows, whereas the top 5 down-regulated genes averaged a –3.3-fold decrease between groups. The top up-regulated genes included lysozyme (*LYZ*), cystatin B (stefin B) (*CSTB*), glycoprotein (transmembrane) nmb (*GPNMB*), cathepsin K (*CTSK*), and beta–1,4-N-acetyl-galactosaminyl transferase 3 (*B4GALNT3*) (Table 5). Lysozyme showed the highest transcriptional increase (log_2_ fold-change is 4.5). The top down-regulated genes in the ileocecal valve tissue were granulysin-like (*GNLY*), membrane metallo-endopeptidase (*MME*), maltase-glucoamylase (alpha-glucosidase) (*MGAM*), phospholipase A2, group IIA (*PLA2G2A*), and an uncharacterized protein (Table 5). *GNLY*, antimicrobial peptide produced by the host to control microbial infections, was also one of the most down-regulated genes in the comparison between the clinical and uninfected control animals. This gene was highly down-regulated in tissue from clinical animals, but there was no difference in the level of expression between the uninfected control and subclinical animals.

**Table 5:** Top regulated genes between clinical and subclinical animals.

Gene ID	Description	Log_2_ Fold Change	P-value	FDR
ENSBTAG00000026779	lysozyme (LYZ)	4.54	1.88E-20	2.28E-16
ENSBTAG00000018189	cystatin B (stefin B) (CSTB)	4.12	5.01E-16	3.04E-12
ENSBTAG00000000604	glycoprotein (transmembrane) nmb	3.98	2.09E-12	3.16E-09
ENSBTAG00000021035	cathepsin K (CTSK)	3.92	5.46E-15	1.66E-11
ENSBTAG00000008553	beta-1,4-N-acetyl-galactosaminyl transferase 3 (B4GALNT3)	3.44	1.43E-13	3.47E-10
ENSBTAG00000013039	Uncharacterized protein	-2.81	1.28E-03	5.78E-02
ENSBTAG00000005759	phospholipase A2, group IIA (platelets, synovial fluid) (PLA2G2A)	-2.92	5.50E-06	9.96E-04
ENSBTAG00000046152	maltase-glucoamylase (alpha-glucosidase)	-2.96	5.08E-04	3.18E-02
ENSBTAG00000002075	membrane metallo-endopeptidase	-3.60	8.53E-06	1.33E-03
ENSBTAG00000031828	granulysin-like (LOC616323) (GNLY)	-4.12	1.84E-04	1.41E-02

Pathway analysis identified several pathways that are involved in the control of infectious disease (Fig 4C). The lysosomal genetic pathway was the top dysregulated pathway identified (Table 6). There are 13 total genes annotated as cathepsins in the *Bos taurus* genome, and of those, 7 are identified as DE genes in this group. There were several glycosidases, sulfases, and lipases that were also classified as DE. All of these DE genes in the lysosomal pathway are up-regulated in the clinical animals, with the exception of one sulfase, arylsulfatase B (*ARSB*), which was down-regulated approximately 2-fold in the clinical animals.

**Table 6:** Regulation of DE genes involved in relevant pathways between clinical and subclinical animals.

Gene ID	Description	Log_2_ Fold Change	P-value	FDR
**Lysosomal pathway**				
ENSBTAG00000021035	cathepsin K (CTSK)	3.92	5.46E-15	1.66E-11
ENSBTAG00000000133	CD68 molecule (CD68)	2.98	1.77E-11	1.95E-08
ENSBTAG00000007622	cathepsin D (CTSD)	1.97	8.05E-06	1.28E-03
ENSBTAG00000017465	glucosamine (N-acetyl)-6-sulfatase (GNS)	1.63	2.40E-08	1.42E-05
ENSBTAG00000011100	cathepsin C (CTSC)	1.53	4.58E-07	1.63E-04
ENSBTAG00000018784	cathepsin Z (CTSZ)	1.52	7.22E-08	3.24E-05
ENSBTAG00000012442	cathepsin B (CTSB)	1.46	3.33E-05	4.09E-03
ENSBTAG00000010992	cathepsin H (CTSH)	1.46	6.36E-05	6.07E-03
ENSBTAG00000003636	lipase A, lysosomal acid, cholesterol esterase	1.35	1.96E-04	1.49E-02
ENSBTAG00000011257	N-acylsphingosine amidohydrolase (acid ceramidase) 1 (ASAH1)	0.96	7.08E-05	6.50E-03
ENSBTAG00000027337	cathepsin A (CTSA)	0.94	2.10E-04	1.56E-02
ENSBTAG00000015512	hexosaminidase B (beta polypeptide) (HEXB)	0.92	3.17E-05	3.96E-03
ENSBTAG00000019256	galactosidase, alpha	0.85	6.17E-04	3.65E-02
ENSBTAG00000008341	arylsulfatase B (ARSB)	-0.99	8.22E-04	4.41E-02
**Complement and Coagulation Pathway**				
ENSBTAG00000020872	complement component 5a receptor 1 (C5AR1)	2.30	2.17E-06	5.48E-04
ENSBTAG00000007101	coagulation factor III (F3)	2.22	1.05E-15	4.25E-12
ENSBTAG00000013125	plasminogen activator, urokinase receptor (PLAUR)	2.13	1.57E-05	2.14E-03
ENSBTAG00000006984	CD55 molecule, decay accelerating factor for complement (CD55)	1.97	2.77E-05	3.65E-03
ENSBTAG00000005947	plasminogen activator, urokinase (PLAU)	1.52	1.86E-06	4.89E-04
ENSBTAG00000007411	coagulation factor VII (serum prothrombin conversion accelerator) (F7)	-1.42	2.24E-04	1.65E-02
ENSBTAG00000017714	complement component 4 binding protein, beta	-2.24	4.42E-04	2.86E-02
**NOD-like Receptor Signaling Pathway**				
ENSBTAG00000038235	nucleotide-binding oligomerization domain containing 1 (NOD1)	2.05	5.44E-08	2.64E-05
ENSBTAG00000019716	interleukin 8 (IL8)	1.77	4.59E-05	4.97E-03
ENSBTAG00000003326	NLR family, apoptosis inhibitory protein	1.74	2.99E-06	6.49E-04
ENSBTAG00000037811	chemokine (C-C motif) ligand 2 (CCL2)	1.61	8.25E-04	4.41E-02
ENSBTAG00000020936	nucleotide-binding oligomerization domain containing 2 (NOD2)	0.94	4.42E-04	2.86E-02
ENSBTAG00000010007	mitogen-activated protein kinase 13 (MAPK13)	0.87	8.50E-04	4.52E-02
**Leukocyte Transendothelial Migration**				
ENSBTAG00000020676	matrix metallopeptidase 9 (MMP9)	2.24	5.69E-05	5.57E-03
ENSBTAG00000019953	cytochrome b-245, beta polypeptide (CYBB)	2.20	2.79E-06	6.15E-04
ENSBTAG00000026278	claudin 4 (CLDN4)	1.97	2.01E-10	2.03E-07
ENSBTAG00000008004	neutrophil cytosolic factor 2	1.70	5.30E-04	3.22E-02
ENSBTAG00000010303	intercellular adhesion molecule 1 (ICAM1)	1.58	8.20E-06	1.29E-03
ENSBTAG00000017060	integrin, beta 2 (complement component 3 receptor 3 and 4 subunit) (ITGB2)	1.22	3.73E-05	4.43E-03
ENSBTAG00000010007	mitogen-activated protein kinase 13 (MAPK13)	0.87	8.50E-04	4.52E-02

Other immunological related pathways include the complement and coagulation cascades, NOD-like receptor signaling, and leukocyte transendothelial migration (Table 6). There are a total of 7 DE genes of the 71 total genes in the complement and coagulation pathway. Five of these genes are up-regulated by at least 3-fold in the clinical animals. The other two genes are down-regulated by at least 3-fold. These pathways point to an immune system actively fighting an infection and suggest complement is important to that goal.

## Discussion

Transcriptome analysis of the host response to bacterial infections has enhanced our understanding of the cellular mechanisms that are involved in the progression of diseases. For many years this field was dominated by the use of microarrays to identify gene transcription on a comparative basis. Although notable progress was made in our understanding of the mechanisms involved in disease progression of paratuberculosis [21, 22], microarrays have several limitations. Some of these limitations include a prior knowledge of the genome and annotated genes for probe design, constrained ranges of gene expression, and indirect gene expression quantification based upon signal intensity. RNA-seq overcomes many of these limitations and gives a fully quantitative and qualitative view of the transcriptomics of the organism being studied. There have been numerous *in vitro* transcriptional studies performed on the macrophage response to mycobacterial infections [23–25]. Although transcriptional studies have been performed on tissues from animals infected with MAP as well, these studies were more focused on genes involved in host immunity [26–28]. To date, only one additional study has provided comprehensive gene expression profiling of responses to MAP infection [10]. However, in that study the goal was to ascertain early events in MAP infection so a calf ligated loop infection model was used. To our knowledge, the present study is the first to use high-throughput RNA-seq to examine the transcriptomes in ileocecal valve tissue from dairy cows that were naturally infected with MAP. The animals in this study were at varying stages of disease to identify transcriptional changes and cellular pathways that may be critical to the progression of paratuberculosis from an asymptomatic stage to a more advanced clinical state.

In the comparison of clinical animals with uninfected control animals, there were 823 DE genes identified. Several of the most highly regulated genes in this comparison are involved in the immune response to pathogens, namely immunoglobulin superfamily member 23 (*IGSF23*), granulysin-like (*GNLY*), Fc receptor-like A (*FCRLA*), and CD79b, with the first two genes demonstrating up regulation and the latter two down regulation in clinical cows. Little is known about the protein IGSF23, but other proteins in this family are found on the cell surface and act as a co-receptors or co-stimulators of cell surface proteins on immune cells, such as T- and natural killer (NK) cells, as well as extracellular matrix proteins [29]. Granulysin-like acts as an antimicrobial peptide that is produced by activated cytotoxic, NK, and γδ T-cells to control microbial infections, and is particularly important in controlling mycobacterial infections [30, 31]. Granulysin levels in patients with *M*. *tuberculosis* are lower in infected patients than healthy controls. Mycobacteria may be actively down regulating granulysin, further studies will need to be done to identify a mechanism.

Fc receptor-like proteins have recently been described as being primarily expressed on B-cells and are involved in immunoregulatory functions [32]. The *CD79b* gene encodes the Igβ protein of the B-cell receptor, which is required for the function of the B-cell receptor [33]. It has been shown that there is a prolific B cell response to MAP early in the infection that wanes over time, resulting in weaker responses to mitogens, possibly due to the reduction in the expression of *CD79b* (reviewed in [34]).

Changes in the regulation of genes in the TCR signaling pathway in the ICV of clinical animals is a significant finding. Previous studies have documented a reduction of T_H_1 cell-mediated immunity, with decreases in numbers of CD4 T cells and reductions in cytokines produced by T_H_1 cells, such as IFN-γ [35, 36]. The down regulation of CD3δ, a TCR co-receptor that is critical for the development of T-cells as well as the activation of the TCR, noted here may be a key mechanism for the loss in cell-mediated immunity that is associated with clinical paratuberculosis [37]. CD40L (also known as CD154), is a member of the TNF superfamily that is primarily expressed on activated T-cells as well as other cell types [38]. Reduced expression of CD40L in the ICV of clinical cows is interesting as the binding of CD40 on APCs with CD40L on activated T-cells initiates a proinflammatory response. Previously, expression of CD40 and CD40L on PBMCs was increased in cows naturally infected with MAP, regardless of stage of infection, upon stimulation of cells with live MAP [39]. Since CD40L is expressed primarily on activated CD4+ T cells, it is likely that this represents a decreased presence of this cell type within the ICV as reduced numbers of CD4+ T cells have been observed in cattle with clinical disease [40]. The protein encoded by the *PTPRC* gene is a protein tyrosine phosphatase (also known as leukocyte common antigen (LCA)) that is present on the cell surface of leukocytes [41]. It has been shown to be a regulator of maturation and activation pathways in leukocytes by regulating the activity of many Src family kinases [42]. Activation of these kinases can result in the activation of phospholipase D in macrophages, which promotes the phagocytosis of bacteria and killing of mycobacteria [43]. The gene product of *PD-1* is a cell surface protein that is part of the immunoglobulin superfamily. This protein is involved in the regulation of T-cell function by promoting self-tolerance, preventing auto-immunity, and inducing apoptosis [44]. Increased levels of PD-1 have been shown to lead to decreases of IL-17 and IL-23 production in patients with *M*. *tuberculosis* [45]. The proinflammatory cytokine, IL-17, also plays a role in MAP infection after a T-helper type 17 response gets established [46]. Expression levels of IL-17 in this experiment were below the low expression threshold, and, therefore, were not included in the statistical analysis.

Key genes within the apoptosis pathway that were differentially expressed in the clinical animals included increased expression of the death receptor tumor necrosis factor receptor superfamily, member 1A (*TNFRSF1A*), thereby contradicting the idea that MAP suppresses apoptosis [47]. This is one of the major receptors of TNF-α and can lead to increased inflammation, apoptosis, or necrosis depending upon the downstream pathways that are activated. Additionally, IL-1β expression was up-regulated in the ICV from clinical cows. Expression of the IL-1β gene is induced by NF-κB and leads to recruitment of macrophages and neutrophils to the site of infection with consequential increases in local inflammatory responses, commonly associated with clinical paratuberculosis [48]. Interestingly, two major genes of the B-cell receptor (BCR) pathway, CD79a and CD79b, which encode the Igα and Igβ proteins, were down-regulated in clinical animals [49]. Although the number of B cells in peripheral blood has been shown to increase in animals with clinical disease, the proliferative responses of the B cells to mitogens and antigens is reduced [50, 51].

The MAPK signaling cascade is a series of conserved serine/threonine kinases that relay extracellular signals (often from toll-like receptors) to transcription factors that mediate the expression of key immune mediators such as inflammatory chemokines and cytokines IL-1, IL-10, IL-12, and TNF-a genes. Genes encoding members of two of the three tiers of the MAPK signaling pathway were differentially expressed in this study. MAPK6 (also called ERK3) and MAPK13 (also called p38δ) were up-regulated, whereas MAP3K14 (also known as NIK) was down-regulated in the clinical animals. Also, the genes that encode the transcription factors JUN and FOS were transcriptionally up-regulated. These data are consistent with previous studies that have shown the MAPK signaling pathway is modulated during mycobacterial infections [52, 53]. The clinical significance of the up regulation of the MAPK kinases in macrophages in advanced mycobacterial diseases is an increase in phagosome acidification, NO2 production and apoptosis. However, ERK3 and p38 have been shown to induce IL-10 expression, which suppresses macrophage and Th1-type cell activation, a possible weakness in the host response the pathogen can exploit [54].

The number of genes that were differentially expressed between subclinical animals and uninfected controls was similar to the number of DE genes between subclinical and clinical cows. However, pathway analysis revealed that very few of the genes between these two comparison groupings were shared. Metabolic pathways were most notable for the subclinical-control uninfected animal comparison with only 5 or fewer DE genes within each pathway. These results would suggest that while there are differences in gene expression, these differences do not affect any major pathway in a significant manner, particularly those controlling immunological function. This finding is consistent with other published literature showing there are very few differences between subclinical and uninfected control animals [21]. This is disappointing as one application for DE gene expression would be as potential biomarkers to detect early infection in the host. Studies that have defined metabolic biomarkers in serum of experimentally infected cattle have demonstrated that infection results in decreased energy and increased protein turnover [55], as well as vitamin D binding protein precursor, transthyretin, retinol binding protein, and cathelicidin [56]. It was also observed that indoleamin 2, 3-dioxygenase, an enzyme involved in killing of intracellular pathogens, was correlated with lesion severity and pathogen load in MAP-infected sheep [57]. Perhaps with deeper sequencing in further experiments, potential biomarkers would be ascertained. However, one must be cautionary in the use of metabolic biomarkers that may be dysregulated nonspecifically during infection.

In contrast, the comparison between clinical and subclinical animals identified differences in some major pathways related to immune function, including the lysosomal, complement and coagulation, NOD receptor signaling, leukocyte transendothelial migration, and several chemokine pathways. The up-regulation of IL-8, a chemokine responsible for migration of neutrophils to the site of infection, as well as induction of neutrophil phagocytosis, has not been previously been shown. Although neutrophils are not recognized as a major immune cell in host defense against MAP infection, they are recruited to disease lesions, caused by MAP infection, in the gut [3]. Studies have shown that matrix metallopeptidases (MMPs) are up-regulated soon after infection with mycobacteria, including MAP, leading to several inflammatory tissue changes [58]. MMPs are important in influencing immune cell migration as well as controlling extracellular signaling molecules, such as cytokines, chemokines, and growth factors. In the present study MMP-3, -9, -13, -14, and -19 genes were up-regulated in ICV tissue of clinical animals indicating that the tissue may be in a state of heightened inflammation, with increased recruitment of neutrophils as well as up regulation of lysosomal peptidases.

The gene products of NOD1 and NOD2 are key cellular receptors that recognize intracellular pathogens and have been shown to be important in the recognition of MAP in epithelial cells early in infection [59]. Expression of pattern recognition receptors, including NOD2 were previously shown to be up-regulated in the terminal ileum of paucibacillary and multibacillary sheep with symptomatic paratuberculosis [60]. In the present study, expression of both NOD1 and NOD2 were up-regulated in tissue from clinical animals. The additional up regulation (≥ 2-fold) of cathepsins in clinical disease provides further evidence that animals in advanced stages of infection are still able to maintain a highly reactive innate immune response with cellular-pathogen engagement and initiation of key intracellular pathways for cellular activation.

## Conclusions

The host global gene expression analysis of MAP infection in a prime target organ of a clinically relevant animal host can provide great insights into the progression of this chronic and debilitating disease, particularly during the transition from subclinical to clinical disease. Our analyses on the gene expression of animals in two defined disease states adds to the understanding that MAP is able to prevent detection by the host immune system until the late stage of clinical disease, when major indications of severe inflammation are present. In the present study, several key immune pathways remained stable in the subclinical animals, but were modulated in tissue from the clinical animals, both in comparison to uninfected control animals as well as animals with subclinical infection. The present approach of using RNA-seq on tissues from naturally infected animals demonstrates the importance of viewing the disease from a holistic approach to identify genes and pathways that can be investigated further in the future to aid in better diagnostic tools and vaccines.
